# Whole-Transcriptome Analysis Sheds Light on the Biological Contexts of Intramuscular Fat Deposition in Ningxiang Pigs

**DOI:** 10.3390/genes15050642

**Published:** 2024-05-19

**Authors:** Zhao Jin, Hu Gao, Yawei Fu, Ruimin Ren, Xiaoxiao Deng, Yue Chen, Xiaohong Hou, Qian Wang, Gang Song, Ningyu Fan, Haiming Ma, Yulong Yin, Kang Xu

**Affiliations:** 1 College of Animal Science and Technology, Hunan Agricultural University, Changsha 410128, China; jz2725552543@163.com (Z.J.); gaohu_20190008@163.com (H.G.); fuyw2020@163.com (Y.F.); wangq0130@163.com (Q.W.); songgang19971109@163.com (G.S.); fny1022@163.com (N.F.); mahaiming2000@163.com (H.M.); yinyulong@isa.ac.cn (Y.Y.); 2 Key Laboratory of Agroecological Processes in Subtropical Region, Institute of Subtropical Agriculture, Chinese Academy of Sciences, Changsha 410125, China; ruimin.ren@hunau.edu.cn (R.R.); dengxiaoxiao@isa.ac.cn (X.D.); 18837025618@126.com (Y.C.); 17865669807@163.com (X.H.); 3 Guangdong Laboratory for Lingnan Modern Agriculture, Guangzhou 510642, China; 4 Hunan Provincial Key Laboratory of the Traditional Chinese Medicine Agricultural Biogenomics, Changsha Medical University, Changsha 410219, China

**Keywords:** pig, meat quality, intramuscular fat, mRNA, non-coding RNA

## Abstract

The quality of pork is significantly impacted by intramuscular fat (IMF). However, the regulatory mechanism of IMF depositions remains unclear. We performed whole-transcriptome sequencing of the longissimus dorsi muscle (IMF) from the high (5.1 ± 0.08) and low (2.9 ± 0.51) IMF groups (%) to elucidate potential mechanisms. In summary, 285 differentially expressed genes (DEGs), 14 differentially expressed miRNAs (DEMIs), 83 differentially expressed lncRNAs (DELs), and 79 differentially expressed circRNAs (DECs) were identified. DEGs were widely associated with IMF deposition and liposome differentiation. Furthermore, competing endogenous RNA (ceRNA) regulatory networks were constructed through co-differential expression analyses, which included circRNA-miRNA-mRNA (containing 6 DEMIs, 6 DEGs, 47 DECs) and lncRNA-miRNA-mRNA (containing 6 DEMIs, 6 DEGs, 36 DELs) regulatory networks. The circRNAs sus-TRPM7_0005, sus-MTUS1_0004, the lncRNAs SMSTRG.4269.1, and MSTRG.7983.2 regulate the expression of six lipid metabolism-related target genes, including *PLCB1*, *BAD*, and *GADD45G*, through the binding sites of 2-4068, miR-7134-3p, and miR-190a. For instance, MSTRG.4269.1 regulates its targets *PLCB1* and *BAD* via miRNA 2_4068. Meanwhile, sus-TRPM7_0005 controls its target LRP5 through ssc-miR-7134-3P. These findings indicate molecular regulatory networks that could potentially be applied for the marker-assisted selection of IMF to enhance pork quality.

## 1. Introduction

Pork is the world’s second most consumed meat, with increasing focus being placed on pork quality. The meat’s tenderness, flavor, and juiciness are greatly affected by intramuscular fat (IMF) content [1]. Regarding sensory properties and nutritional value, meat quality is primarily dependent on the IMF levels and fatty acid constitution [2]. The IMF includes the cholesterol, triglyceride, and phospholipid content in the muscle [3] and is the final step of fat deposition in the body [4]. Volatile lipid oxidation products are produced primarily by fatty acids during the cooking process, which contributes to meat flavor via the Maillard reaction. Therefore, it is necessary to inquire into the genetic mechanisms of IMF deposition to improve meat quality.

Non-coding RNAs, including circular RNA (circRNA), long non-coding RNA (lncRNA), and microRNA (miRNA), regulate protein-coding genes and play crucial roles in diverse biological processes. The construction of competing endogenous RNA (ceRNA) networks, which involve circRNA, lncRNA, miRNA, and mRNA, can enable the further understanding of complex molecular mechanisms [5,6,7]. miR-34a inhibits porcine intramuscular preadipocyte adipogenesis through Erk signaling pathways by targeting PDGFRα [8]. LncRNA IMFNCR promotes adipocyte differentiation by upregulating *PPARG* expression via miR-128-3p and miR-27b-3p [9]. CircRNAs regulate gene expression in IMF deposition by adsorbing miRNAs. For instance, circRNA06424 modulates the level of peroxisome proliferator-activated receptor delta (*PPARD*) by competitively combining with miRNA, thereby regulating subcutaneous fat and IMF [10]. However, there is little research on the molecular mechanisms that regulate IMF deposition in Ningxiang pigs.

Some studies focused on the differences between high and low IMF levels in the same breed. Previous studies have compared the genetic profiles of Laiwu pigs with different IMF levels, and the results indicated that the different IMF levels between individual pigs may be due to DE lncRNAs and mRNAs associated with lipid droplets and fat deposition [11]. By comparing the whole transcriptome expression profiles of the longest dorsal muscles of pigs with high and low IMF contents in Yorkshire pigs, the results have shown that IMF fat formation in Yorkshire pigs is regulated by the ceRNA network formed by functional lncRNAs and circRNAs [12]. The Ningxiang pig is one of China’s most outstanding native pig breeds, renowned for its high and evenly distributed intermuscular fat content, tender meat, and slender muscle fibers [13]. It is recognized as an important nationally livestock gene pool. Ningxiang pigs have an excellent genetic basis in meat quality, which makes them an ideal model for studying IMF.

In this study, we identified differentially expressed genes (DEGs) in high and low IMF levels of Ningxiang pigs by whole-transcriptome analysis, co-expression network analysis, and functional enrichment. We further explored their regulatory roles as competing endogenous RNAs (ceRNA) in lipid metabolism. These results may contribute to improving the genetic and molecular characteristics of pork, as well as providing insights into human health concerns and obesity.

## 2. Materials and Methods

### 2.1. Sample Separation

In this study, two hundred healthy castrated male Ningxiang pigs were selected from the Ningxiang pig population. The pigs are maintained under controlled conditions of temperature (18–25 °C), humidity (60 ± 5%), and ventilation, with consistent feeding management practices. All experimental pigs were obtained from the Dalong Livestock Co. Ltd., in Changsha, China, and all pigs were provided ad libitum access to water and were fed the same diet until they reached 180 days of age in the study. The nutritional level of the diets (Table S1) was assessed according to the Pig Nutrient Requirements of China (GB/T 39235-2020). The pigs were weighed and then butchered in Chu Weixiang commercial abattoirs in Ningxiang City, Hunan Province. Following slaughter, the guts, intestines, heads, and blood were removed (with the kidneys and suet retained), and the carcass weight was recorded 1 h after slaughter (average carcass weigh: 50 ± 5.5 kg). For comparison, the samples were collected from the same part of the longissimus dorsi muscle (LDM) (10th to 11th ribs) and preserved at −80 °C.

### 2.2. Indicator Determination

After the removal of fascia, blood vessels, and connective tissue, the LDM sample was ground using a meat grinder. Subsequently, the Soxhlet petroleum–ether extraction method was applied to measure the IMF levels of the 200 LDM samples [14]. Two extreme groups of individuals were selected according to the IMF content (%): the high (5.1 ± 0.08) and low (2.9 ± 0.51) IMF groups (4 individuals per group). Fatty acid methyl ester was extracted based on the GB_5009.168-2016 and Floch [15] method and analyzed by gas chromatography. A 0.5 g quantity of the dry sample was added to 2 mL of a 1:1 mixture of benzene and petroleum ether, sealed, and soaked for 24 h. A 2 mL volume of KOH–methanol solution (0.4 mol/L) was added for rapid methyl esterification. The mixture was shaken thoroughly and was allowed to rest for 2 h to allow for stratification. Gradually distilled water was added along the wall of the test tube until the methanol solution volume reached 10 mL. It was allowed to sit until it was clarified, typically around 30 min. The sample was centrifuged at 10,000 rpm for 10 min to obtain 100 μL of supernatant. Then, the fatty acid methyl esters were analyzed using a gas chromatograph GC6890N (Agilent Technologies, CA, USA), and the resulting supernatant was diluted with hexane. The fatty acids content was determined using a gas chromatograph (Agilent 7890A, Santa Clara, CA, USA). The gas chromatography conditions were as follows: the column used was an SP-2560 (100 mm × 0.25 mm, 0.20 μm) capillary column and high-purity nitrogen served as the carrier gas. The heating procedure was as follows: an initial temperature of 120 °C was maintained for 5 min, then increased to 170 °C at a rate of 2 °C/min, maintained for 15 min, and finally increased to 235 °C at a rate of 2 °C/min, and maintained at this temperature for 10 min. The sample inlet temperature was 260 °C, the flame ionization detector (FID) temperature was 280 °C, and the injection volume was 1 μL. Fifteen fatty acids were detected in total, with each fatty acid signal was quantified as a percentage of the total fatty acid methyl esters (Table S2). Each sample was determined twice.

### 2.3. RNA Preparation

Total RNA was isolated from the LDM of Ningxiang pigs by Trizol reagent (Invitrogen, Carlsbad, CA, USA) following the manufacturer’s instructions. Agarose gel electrophoresis was performed to determine the completeness of the RNA. The purity and concentration of RNA was detected by Nanodrop (OD260/280 ratio 1.8 to 2.2) and Qubit fluorometer (≥500 ng/μL) [16]. In addition, ribosomal RNA was extracted from each sample by the Ribo-Zero Gold Kit (Epicentre, Madison, WI, USA). Two libraries were generated for whole-transcriptome sequencing (WTS): one small RNA library generated for miRNA analysis and a ribosome-removed library generated for lncRNA/circRNA analysis. The strand-specific library and small RNA library were sequenced on the HiSeq 3000 platform by PE150 and SE50, respectively. Deep sequencing was performed by Shanghai Genergy Bio Technology Co., Ltd. (Shanghai, China). Fast QC software (v0.11.5) was utilized to check the quality of the data obtained.

### 2.4. Data Analysis

The sequencing data was cleaned utilizing Trim Galore (v0.4.2) to remove splices and poor-quality fragments. For quality assurance, the preprocessed data was examined by Fast QC (v0.11.5). The clean reads were compared to the Porcine reference genome (Scrofa11.1) using STAR (version 2.5.3a) with default parameters. Transcripts were constructed by String Tie (v1.3.1c) and compared to reference genomes in the Ensembl database using Cuff compare, retaining transcripts containing multiple exons greater than 200 bp in length. To determine whether these transcripts were coding-competent, three software packages were used for analysis, namely, PLEK (1.2), CNCI (1.2.2), and CPAT (1.2.2). CIRC explorer2 was used to predict the cyclic RNA. Thereafter, mRNA and lncRNA transcript expression was calculated using FPKM (Fragments Per Kilobase of transcript per Million fragments mapped). BSRP (Back Spliced Reads Per Million Mapping Reads) was employed to analyze the expression of cyclic RNA. The inclusion criteria for DEMIs, DECs, and DELs were |log_2_ fold-change (FC)| ≥ 1 and *p*-value ≤ 0.05. The inclusion criteria for DEGs with FDR ≤ 0.05 and |log_2_ fold-change (FC)| ≥ 1 (Table S3).

### 2.5. Enrichment Analysis

The DEGs were annotated with GO via ClusterProfiler to describe the function of the differentially expressed genes (in combination with GO annotation results). The CC, MF, and BP annotations of differentially expressed genes were identified by the Fisher algorithm [17]. Finally, miRbase database was analyzed via the KEGG Orthology database [18].

### 2.6. CeRNA Network Construction

The interactions of miRNAs and lncRNAs were forecasted using the Start Base database, while the connection between mRNAs and miRNAs was predicted using miRanda (v3.3a). Then, the discovered miRNAs were crossed with the predicted miRNAs from circRNA or lncRNA to establish the ceRNA networks. According to miRNAs correlated with DECs and DELs individually and collectively, ceRNA networks associated with DECs and DELs were constructed [19]. The coregulation network was displayed utilizing Cytoscape (version 3.6.1).

### 2.7. RT-qPCR (Real-Time Quantitative PCR)

RT-qPCR was performed on four RNA samples from each of the two groups to verify the accuracy of the RNA-seq data. Using the Prime Script™ RT reagent Kit with gDNA Eraser (Takara, Dalian, China), total RNA was reverse transcribed into cDNA. Specific primers were devised with Primer 5.0 and were synthesized by Beijing Tsingke Biotech Co., Ltd., Beijing, China (Table 1). The qPCR was conducted by mixing 5 µL of 2× TB Green Premix Ex Taq II (Takara), 0.5 µL of each primer (forward and reverse), 200 ng of cDNA template, and RNase-free water up to 10 µL and run on a Light Cycler 384 real-time PCR system (Roche, Basel, Switzerland) with the following program: Stage 1: 95 °C for 30 s; Stage 2: 95 °C for 5 s and 60 °C for 30 s for 40 cycles; Stage 3: 95 °C for 5 s, 60 °C for 1 min and 95 °C for 5 s; and Stage 4: 50 °C for 30 s. The 2^−∆∆CT^ approach was utilized, and quantitative analysis was carried out. Glyceraldehyde-3-phosphate dehydrogenase (*GAPDH*) was used for lncRNA, β-actin for mRNA and circRNA, and *U6* for miRNA [20].

### 2.8. Statistical Analysis

The *t*-test was utilized to analyze the data. Four biological replicates were performed for each sample. The mean and standard error of every experiment’s data were displayed, and statistically significant *p* values of less than 0.05 were taken into account. The plots of RT-qPCR and RNA-seq were created via GraphPad Prism 9.0.

## 3. Results

### 3.1. Phenotypic Data of the LDM in Different Intramuscular Fat Content Groups

**Figure 1:**
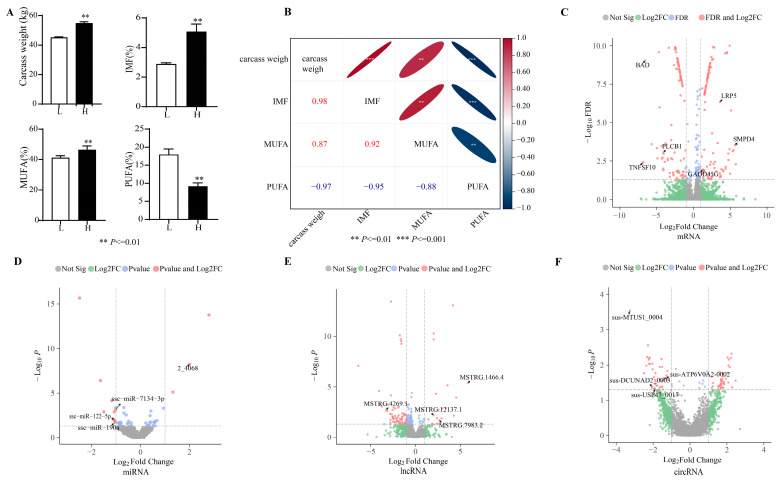
The fat content of the LDM samples and volcano plot. (**A**) The carcass weight, the content of intramuscular fat (IMF), and the monounsaturated fatty acid (MUFA) and polyunsaturated fat (PUFA) levels between the low- and high-IMF groups and (**B**) correlation analysis. The volcano plot displaying the differentially expressed genes (DEGs) (**C**), differentially expressed miRNAs (DEMIs) (**D**), differentially expressed lncRNAs (DELs) (**E**), and differentially expressed circRNAs (DECs) (**F**), including upregulated and downregulated genes in the two groups (low-IMF vs. high-IMF groups).

The IMF content was significantly higher in pigs with a high carcass weight compared to those with a low carcass weight. The monounsaturated fatty acid (MUFA) content was significantly higher in the group with a high IMF level compared to the group with a low IMF level, whereas polyunsaturated fats (PUFAs) were lower (Figure 1A). Correlational analysis showed a close positive correlation among IMF and MUFA, whereas PUFA was significantly negatively correlated with IMF (Figure 1B).

### 3.2. Data of Sequencing

To evaluate the genes related to IMF deposition, the LDM was collected, and the total mRNAs, lncRNAs, circRNAs, and miRNAs were analyzed using WTS. RNA sequencing of the eight samples yielded an average of 108.62 million clean reads, with the Q30 reads exceeding 92.50%. All eight samples had 94.3–97.1% of reads aligned to the reference genome, indicating that the data quality was acceptable for further analysis. In addition, the average number of clean reads for small RNA-Seq libraries was 23.47 million. A total of 97.15% to 98.16% of reads were matched to the pig reference genomes, while 91.33–94.08% of reads were uniquely matched to the reference genome miRbase database.

### 3.3. Differentially Expression Genes

Overall, 285 differentially expressed genes (DEGs) were identified among both groups, with 147 downregulated mRNAs and 138 upregulated mRNAs in the high-IMF group (Figure 1C). The results included DEGs concerned with lipid synthesis and metabolism, such as BCL2-associated agonist of cell death (*BAD*, log_2_FC = −6.551), phospholipase C beta 1 (*PLCB1*, log_2_FC = −4.080), LDL receptor-related protein 5 (*LRP5*, log_2_FC = 3.617), and growth arrest and DNA damage-inducible protein gamma (*GADD45G*, log_2_FC = 1.279).

**Figure 2:**
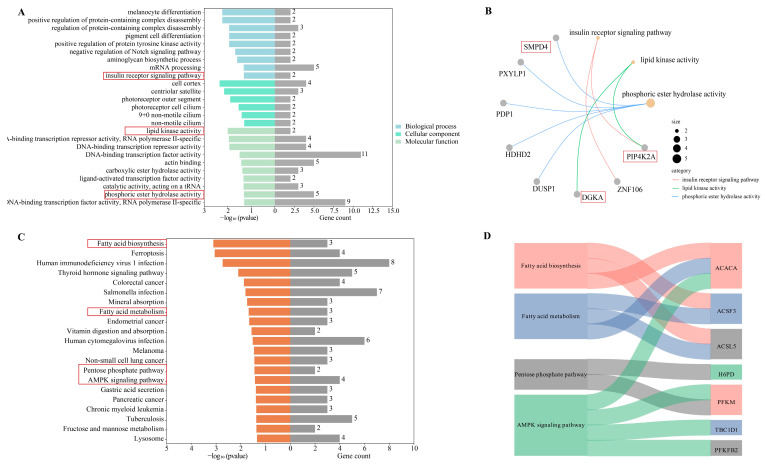
GO annotation and KEGG analysis for DEGs. (**A**) GO annotation of DEGs; (**B**) GO annotation in BP and MF of DEGs; and (**C**,**D**) KEGG analyses of DEGs (The red boxes are genes/entries related to lipid metabolism).

Based on the identified DEGs, GO and KEGG analyses were conducted. The GO annotation results were classified into the cellular components (CCs), molecular functions (MFs), and biological processes (BPs). The BP and MF results were primarily significantly enriched in lipid metabolism, including insulin receptor signaling pathway (GO:0008286), lipid kinase activity (GO:0001727), and phosphoric ester hydrolase activity (GO:0042578) (Figure 2A). Specifically, phosphatidylinositol-5-phosphate 4-kinase type 2 alpha (*PIP4K2A*) and sphingomyelin phosphodiesterase 4 (*SMPD4)* were annotated in BP processes (Figure 2B). Moreover, diacylglycerol kinase alpha *(DGKA)* and *PIP4K2A* were engaged in lipid kinase activity.

The KEGG annotation indicated that the pentose phosphate pathway, the fatty acid biosynthesis, and fatty acid metabolism might serve a critical function in lipid deposition (Figure 2C). Acyl-CoA synthetase long-chain family member 5 (*ACSL5*), phosphofructokinase, muscle (*PFKM*), acetyl-CoA carboxylase alpha (*ACACA*), and others were annotated into these two pathways (Figure 2D).

### 3.4. Differentially Expressed miRNAs

In total, 14 differentially expressed miRNAs (DEMIs) were identified in both groups, with the high-IMF group having 11 downregulated and 3 upregulated miRNAs (Figure 1D). For target gene anticipation of the 12 DEMIs, 359 target genes were obtained.

GO annotation displayed that the target genes were markedly enriched in lipid breakdown, including phosphatidylinositol-3-phosphatase activity (GO:0004438) and lipid digestion (GO:0044241) (Figure S1B). KEGG pathways were mainly enriched in O-glycan biosynthesis, bile secretion, and the rap 1 signaling pathway.

### 3.5. Differentially Expressed LncRNAs and CircRNAs

There were 83 DELs between the two different IMF groups, with 64 downregulated lncRNAs and 19 upregulated lncRNAs in the high-IMF group (Figure 1E). To confirm their potential role in lipid deposition, the target genes of all DELs were anticipated via the cis and trans algorithms. The analysis demonstrated 83 DELs corresponding to 217 target genes. We calculated Pearson correlation coefficients (r > 0.95 or r < −0.95) between the expression levels of 695 lncRNAs and 1143 mRNAs. Then, we performed functional enrichment analysis of lncRNA target genes using the DAVID platform (considering a significance threshold of *p* < 0.05).

**Figure 3:**
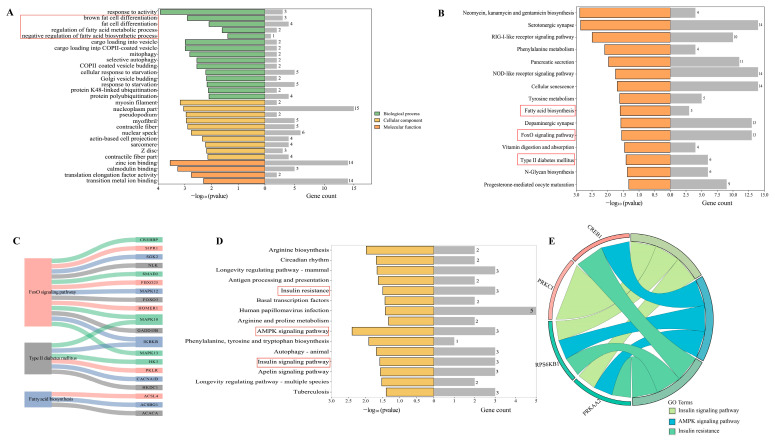
For GO and KEGG annotations, the host genes of DECs and target genes of DELs were analyzed. (**A**) GO functional classification of cis-lncRNA target genes; (**B**) KEGG pathway annotation of trans-lncRNA target genes; (**C**) target genes of trans-lncRNAs involved in differential expression of lipid metabolic pathways; (**D**) KEGG pathway enrichment annotation of DEC host genes; and (**E**) lipid-associated pathway-enriched host genes for DECs (The red boxes are entries related to lipid metabolism).

KEGG pathway annotation revealed that cis-lncRNA target genes were considerably enriched in the mitophagy–animal pathway and protein processing in the endoplasmic reticulum, adherens junction, and endoplasmic reticulum. GO analysis indicated that the cis-lncRNAs target genes were considerably enriched in fatty acids and lipid metabolism, fat cell differentiation (GO:0045444), regulation of fatty acid metabolic process (GO:0019217), and negative regulation of fatty acid biosynthetic process (GO:0045717) (Figure 3A).

Furthermore, KEGG analysis indicated that the trans lncRNAs target genes were remarkably abundant in the Foxo signaling pathway, fatty acid production, and type II diabetes mellitus, all of which are crucial for lipid metabolism (Figure 3B,C). A variety of genes were annotated to these three pathways, including mitogen-activated protein kinase 10 (*MAPK10*), acetyl-CoA carboxylase (*ACACA*), and others (Figure 3D). GO analysis suggested significant enrichment of the lipid metabolism, including phosphatidylinositol monophosphate phosphatase activity (GO:0052744), fatty-acyl-CoA metabolic process (GO:0035337), and phosphatidylinositol phosphate phosphatase activity (GO:0052866).

A total of 20,850 circRNAs were identified between the two different IMF groups. Furthermore, 79 DECs were detected, with 41 upregulated DECs and 38 downregulated DECs in the high-IMF group. (Figure 1F). After circRNA ID conversion by circAtlas, GO and KEGG annotations were applied to reveal the effects of host genes. DECs were remarkably enriched in the AMPK signaling pathway and the insulin signaling pathway (Figure 3D). Moreover, protein kinase AMP-activated catalytic subunit alpha 2 (*PRKAA2*), cAMP-responsive element binding protein 1 (*CREB1*), protein kinase *C iota* (*PRKCI*), and ribosomal protein S6 kinase B1 (*RPS6KB1)* were annotated in these three pathways (Figure 3E). In addition to the catalytic subunit, the regulatory subunit and the AMP-binding subunit constitute the heterotrimeric enzyme AMPK (AMP-activated protein kinase).

### 3.6. CeRNA

**Figure 4:**
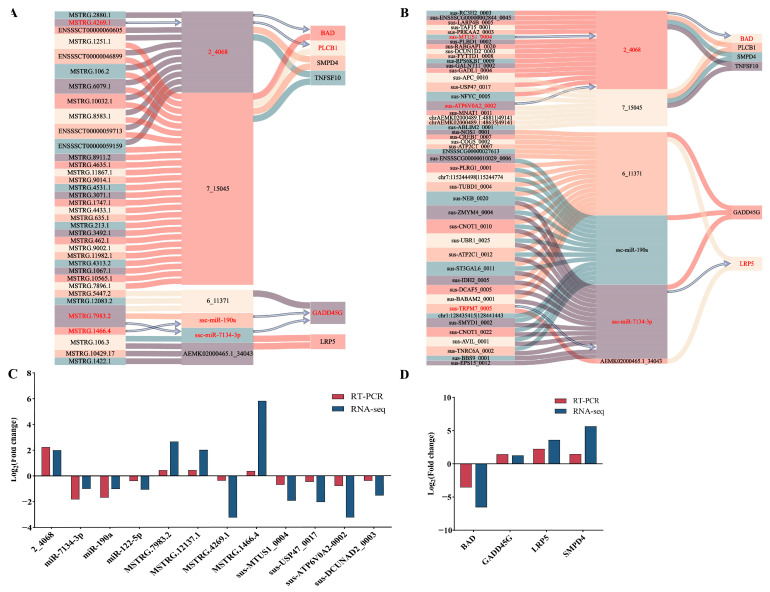
The ceRNA networks. (**A**) Regulatory networks of CircRNA-miRNA-mRNA. (**B**) Regulatory networks of lncRNA-miRNA-mRNA. qPCR analysis to verify the RNA-sequencing results. (**C**) The miRNA, lncRNA, and circRNA expression levels. (**D**) The mRNA expression levels (The main CE network diagrams are shown in red).

Competing endogenous RNA (CeRNA), like circRNA and lncRNA, compete with miRNA to adjust gene levels and thus ensure communication. Analyzing the data of 285 DEGs, 14 DEMIs, 83 DELs, and 79 DECs, a total of 14 miRNA-mRNA regulatory pairs, 47 miRNA-lncRNA regulatory pairs, and 75 miRNA-circRNA regulatory pairs were discovered. The regulatory networks of lncRNA-miRNA-mRNA (including 36 lncRNAs, 6 miRNAs, 6 mRNAs,) (Figure 4A) and circRNA-miRNA-mRNA (including 47 circRNAs, 6 miRNAs, 6 mRNAs) were constructed based on the interactions between the DEGs, DEMIs, DELs, and DECs (Figure 4B). In the two regulatory networks, six shared miRNAs were discovered.

GO and KEGG pathway annotations were performed to determine the role of the target genes in the regulation networks. These genes were markedly enriched in the pathways linked to lipid digestion, including Foxo signaling pathway, wnt signaling pathway, and sphingolipid metabolism (Figure S2A). These genes included growth arrest and DNA damage-inducible protein GADD45 gamma (*GADD45G*), LDL receptor-related protein 5 (*LRP5*), lipase c beta 1 (*PLCB1*), and TNF superfamily member 10 (*TNFSF10*) (Figure S2B).

### 3.7. RT-qPCR Validation of Gene Expression

Ultimately, we randomly selected a total of 16 RNAs (mRNA, lncRNA, miRNA, and circRNA) to confirm the accuracy of the RNA-seq results in this study, which were significant, and DE in the RNA-seq results for RT-qPCR verification. The expression levels of four DECs (sus-MTUS1_0004, sus-USP47_0017, sus-ATP6V0A2-0002, sus-DCUNAD2_0003), four DELs (MSTRG.4269.1, MSTRG.7983.2, MSTRG.1466.4, and MSTRG.12137.1), four DEMIs (miR-122-5p, miR-7134-3p, miR-190a, and 2-4068) (Figure 4C), and four DEGs (*BAD*, *GADD45G*, *LRP5*, and *SMPD4*) (Figure 4D) were verified. The outcomes demonstrated that the RNA-seq data and the change in gene expression in the RT-qPCR results were consistent.

## 4. Discussion

Future research directions are highlighted. Ningxiang pigs are one of the most popular pig breeds in China, having high IMF and unsaturated fatty acid levels [21]. Tenderness, juiciness, and other meat quality characteristics, such as flavor, are influenced by the IMF content. IMF is also reflected in the marbling of the meat, creating a marbled pattern that is one of the main visual criteria for purchasing pork. Therefore, it is important to elucidate the molecular process of IMF deposition in Ningxiang pigs to improve pork quality.

A total of 285 DEGs were identified among the high- and low-IMF groups. For instance, acyl-CoA synthetase family member 3 (*ACSF3*), an essential enzyme for both de novo fatty acid synthesis and oxidation [22]. *ACSF3* combines with thioester and coenzyme A, to activate fatty acids to form acyl coenzyme A, which participates in the metabolism of fatty acids and lipid and binds to ATP [23]. In the present study, the high-IMF group showed higher levels of *ACSF3* expression than the low-IMF group. The higher expression level of insulin-like growth factor 2 mRNA-binding protein 1 (*IGF2BP1*) is linked to better carcass performance in ducks and chickens [24,25]. Total cholesterol and triglyceride levels were decreased in skeletal muscle with high *ACSL5* mRNA levels, and the increase in *ACSL5* expression in muscle tissue was accompanied by a decrease in IMF levels [26,27]. In line with our outcomes, the group with low IMF exhibited higher expression of *ACSL5*.

The first step in the biosynthesis of long-chain fatty acids catalyzed by the *ACACA* gene is very important in energy and lipid metabolism [28]. *ACSL5* plays an essential function in lipid metabolism. *ACSL5* catalyzes the formation of fatty acyl coenzyme a from long-chain fatty acids (C16–C20), which is used in the β-oxidation of fatty acids [29]. As a crucial control enzyme in glycolysis, *PFKM* catalyzes the transformation of fructose 6-phosphate [30]. Diacylglycerol kinases (*DGKs*) convert diacylglycerol to phosphatidic acid via phosphorylation [31]. A low *DGKA* expression was observed in the high-IMF group of Guang ling donkeys, indicating that it might be a candidate gene for regulating lipid deposition [32]. In contrast, the α-subunits α 2 were encoded by *PRKAA2* [33]. As an essential factor of glucolipid metabolism (*GLM*), *AMPK* regulates energy homeostasis in mammals [34]. *CREB1* has been hypothesized to be a vital transcription element involved in lipid metabolism [35]. Evidence suggests that these genes might be critical in the adjustment of lipid deposition.

LncRNAs larger than 200 nt were involved in transcriptional regulation and post-transcriptional regulation [36]. According to sequence data currently available, 83 DELs were detected in the present study. Some of the DEL target genes are crucially linked to IMF deposition. Additionally, peroxisome proliferator-activated receptor beta (*PPARβ*) has a crucial function in adjustments of preadipocyte differentiation and lipid metabolism [37]. LncRNA IMFNCR enhances *PPARG* via competing with miR-27b-3p, thereby regulating the differentiation of intramuscular fat cell [9]. Acyl-CoA synthetase long-chain family member 4 (*ACSL4)* is the target gene of MSTRG.4825.1, MSTRG.4313.2, and MSTRG.213.1. The *ACSL4* gene promotes lipogenesis and may regulate IMF levels in different pig breeds [38]. *ACACA* is the target gene of MSTRG.3071.1 and MSTRG.9636.2 and promotes fatty acid biosynthesis and regulates lipid deposition [39]. Furthermore, *BRCA1* is the target gene of MSTRG.6015.5 and regulates fatty acid synthesis, which is positively correlated to pork IMF content [40]. The above outcomes suggest that these lncRNAs have a vital effect on regulating IMF deposition in pigs.

This study used differential lncRNAs to anticipate target genes in both cis and trans orientations. The GO and KEGG analyses results indicated that the projected target genes were significantly abundant in the type II diabetes mellitus and Foxo signaling pathways, which played an integral function in lipid metabolism. The Foxo signaling pathway affects fat deposition by restraining glycogen accumulation and insulin signal transmission [41]. Further research into their roles would constitute a solid basis for future research.

In the present study, 14 DEMIs were discovered between the high-IMF and the low-IMF groups. miRNAs were shown to have a crucial function in fat deposition, such as pyruvate kinase (*PKM*) participating in upstream pathways in lipid synthesis [42]. *PKM* and miR-122-5p levels were strongly inversely linked [43]. Low levels of miR-122-5p were observed in high-IMF pigs, which is in line with our findings. Moreover, ssc-miR-7134-3p modulates fatty deposition in castrated boars. Microtubule affinity regulating kinase 4 (*MARK4*) contributes to fat accumulation. Castration reduces ssc-miR-7134-3p levels, which leads to increased MARK4 levels and regulates fat accumulation [44]. In agreement with our experiment results, pigs with high IMF exhibited lower levels of miR-122-5p.

In miRNA sponges, circRNA molecules have several miRNA binding sites, which lessen the repressive effects of miRNA and activate gene expression [45]. In this study, 79 DECs were identified. CircRNAs act as an essential regulator of the adipose deposition process. For instance, circSETBP1 enhances IMF deposition by adjusting *CRTCs* with mir-149-5p [46]. Circ-PPARA activated miR-429 and miR-200b in porcine intramuscular adipocytes and enhanced the differentiation, thereby preventing proliferation. This was positively associated with IMF [47]. This was associated positively with the IMF.

Additionally, KEGG and GO analyses showed that target genes of DEMIs and host genes of DECs were considerably enriched in lipid-related pathways, such as the insulin signaling pathway [48,49], AMPK signaling pathway [50,51,52,53], insulin resistance [54,55,56], intestinal lipid absorption, phosphatidylinositol-3-phosphatase activity, and lipid digestion. Phosphorylated AMPK can prevent the expression of essential downstream genes such as acetyl-CoA carboxylase. In addition, activated AMPK can lower lipid deposition by decreasing the production of factors associated with lipid synthesis [57].

Competing endogenous RNAs, circRNAs, and lncRNAs can serve as miRNA sponges during lipid differentiation. For instance, lncRNA4789 can further increase the levels of *FABP3* (miR-204 target gene) and inhibit lipid droplet deposition by modulating miR-381-3P [58]. LncIMF2 enhances lipogenesis in pig intramuscular preadipocytes by regulating MiR-217 [59].

In the current study, ceRNA regulation networks were created employing co-differentially expressed miRNAs, lncRNAs, circRNAs, and mRNA. In total, 36 lncRNAs and 6 mRNAs that crosstalk with one another through 6 miRNAs were identified, as well as 47 circRNAs and 6 mRNAs that interacted through 6 miRNAs. Consistent with earlier findings, IMF accumulation in pigs arises from the expression of genes in equilibrium. Notably, miR-7134-3p mediated the crosstalk between MSTRG.7983.2 and its target *GADD45G*; SSC-miR-190a was involved in the crosstalk between MSTRG.1466.4 and its targets *GADD45G*; MSTRG.4269.1 and its targets *PLCB1* and *BAD* showed crosstalk through 2_4068; sus-TRPM7_0005 and its target *LRP5* showed crosstalk through ssc-miR-7134-3P; sus-MTUS1_0004 and its targets, such as *BAD,* displayed crosstalk through 2_4068. Furthermore, *GADD45G*, *LRP5*, *PLCB1*, and *BAD* are associated with lipid metabolism [60,61,62,63,64]. *LRP5* is a member of the LDLR family and is a multifunctional receptor involved in the maintenance of lipid homeostasis and the typical wnt signaling pathway [65]. *PLCB1* encodes the protein that catalyzes the product of inositol 1,4,5-trisphosphate and diacylglycerol and has a crucial part in lipid metabolism [66]. Moreover, some nodes shared by lncRNA/circRNA-miRNA-mRNA regulatory networks were discovered, such as *PLCB1*, *LRP5*, 2_4068, ssc-7134-3P, and so on. These are likely to be involved in the intricate molecular processes underlying intramuscular fat accumulation.

## 5. Conclusions

In this study, we analyzed the differences in fat accumulation in Ningxiang pig with high and low IMF in LDM tissues. sus-TRPM7_0005, sus-MTUS1_0004, MSTRG.4269.1, MSTRG.7983.2, MSTRG.1466.4, MSTRG.12137.1, 2-4068, miR-7134-3p, miR-190a, and miR-122-5p binding sites acted as sponges to promote the regulation of fat deposition and lipid metabolism by modulating type II diabetes, insulin signaling pathway, and Foxo signaling pathway. However, further studies are needed to explore the profound effects of sus-TRPM7_0005, sus-MTUS1_0004, MSTRG.4269.1, MSTRG.7983.2, MSTRG.1466.4, and MSTRG.12137.1 on the biology of porcine IMF, as well as the role of *SCD5*, *PLCB1*, *BAD*, and *GADD45G* in fat deposition and meat quality. These potential genes and molecular regulatory networks could be utilized in marker-assisted IMF selection to improve pork quality.

## Figures and Tables

**Table 1:** Primer sequences for RT-qPCR.

	RNA	Primer Sequence (5′-3′)	Tm	Product Length (bp)	Genbank ID
mRNA	BAD	F: CTACCAGGCCAGACTCAACC	60	77	XM_021082883.1
		R: AACATGCTCTGGGCTCCAA			
	GADD45G	F: TACGGTTCCAGAAAGCACGG	60	139	NM_001185129.1
		R: GTTGTCGGGGTCCACATTCA			
	SMPD4	F: CTGCTTCTGTTCCAGGTTT	60	92	XM_013981435.2
		R: GATTCCTTGGCATGAGGG			
	LRP5	F: GACCCCTCCCTCTACAACCT	60	83	XM_021082721.1
		R: CGGATGATGTAGGGCCTGTAG			
miRNA	ssc-miR-7314-3P	RT: GTCGTATCCAGTGCAGGGTCCGAGGTATTCGCACTGGATACGACCCGTAT	60	65	
		F: CAGATGCGGAACCTGCGG			
		R: AGTGCAGGGTCCGAGGTATT			
	ssc-miR-190a	RT: GTCGTATCCAGTGCAGGGTCCGAGGTATTCGCACTGGATACGACAGAAGA	60	69	
		F: ACGCGTCTGACTTCCATTCCTT			
		R: AGTGCAGGGTCCGAGGTATT			
	ssc-miR-122-5P	RT: GTCGTATCCAGTGCAGGGTCCGAGGTATTCGCACTGGATACGACACAAAC	60	71	
		F: AACACGCTGGAGTGTGACAA			
		R: CAGTGCAGGGTCCGAGGT			
	2_4068	RT: GTCGTATCCAGTGCAGGGTCCGAGGTATTCGCACTGGATACGACAGAAGA	60	72	
		F: AACACGTGTCTGACTTCCATTC			
		R: CAGTGCAGGGTCCGAGGT			
lncRNA	MSTRG.7983.2	F: ACTCGGCGTTGCTTCTACAG	60	116	
		R: TGAGCTGTGGTGTAGGTTGC			
	MSTRG.4269.1	F: CCAGGCCAAACAACAATCCAG	60	79	
		R: TGTGCCTGAGGGGGTCTTTA			
	MSTRG.1466.4	F: GCTGCACCAGAAGAGGAGTT	60	256	
		R: CGATTCCGAAGGAAGGCAGT			
	MSTRG.12137.1	F: GCCTAGGAACCATGAGGTCG	60	184	
		R: CGGCATATGGAGGTTCCCAG			
CircRNA	sus-USP47_0017	F: ACAGCCAGAGATCCTAGACG	60	79	
		R: AAGACCCTTTCGTGCATCACA			
	sus-DCUNAD2_0003	F: CCTTGCTTCCCAGAGCGTAA	60	83	
		R: CTCTTGCCAGCCCGAGTAAA			
	sus-ATP6V0A2-0002	F: TACACCATCGTGACCTACGC	60	149	
		R: TCCTGCACCAAGTATGCCAA			
	sus-MTUS1_0004	F: ACATCGATGGGATTAGCCCTG	60	108	
		R: AACCGCAGTCAAAGGTCTCA			

## Data Availability

The datasets involved in this study can be found in online repositories. The names of the accession number(s) can be found below: https://www.ncbi.nlm.nih.gov/bioproject/PRJNA1009696 (accessed on 31 August 2023).

## Supplementary Materials

The following supporting information can be downloaded at: https://www.mdpi.com/article/10.3390/genes15050642/s1. Table S1. Ingredient compositions and nutrient levels of diets. Table S2. 15 Fatty acid determination. Table S3. DEGs, DEMIs, DELs, and DECs between high and low IMF. Figure S1. Bioinformatic analysis of DEMIs. Figure S2. KEGG annotation analysis showing differentially expressed genes.
