# Supplementary material for: Whole-Transcriptome Analysis Sheds Light on the Biological Contexts of Intramuscular Fat Deposition in Ningxiang Pigs
Source: Genes (Basel). 2024 May 19;15(5):642. doi: 10.3390/genes15050642 (PMC11121357; doi:10.3390/genes15050642)
Supplement: Supplementary file 1 [file genes-15-00642-s001.zip › Supplementary Figure.pdf]

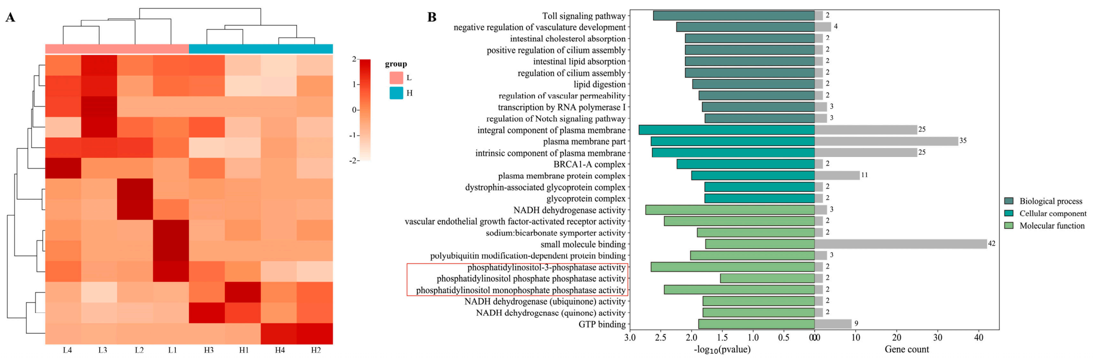

Figure S1. Bioinformatic analysis of DEMIs (a) The Heatmap of DEMIs in the low and high IMF groups; (b) GO enrichment of DEMIs.

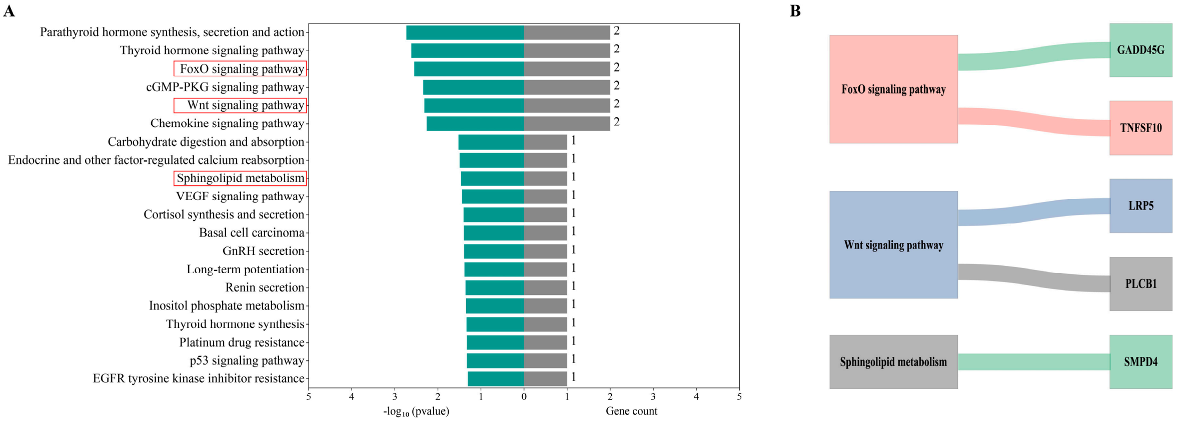

Figure S2. KEGG annotation analysis showing differentially expressed genes. (A) KEGG pathway annotation analysis. (B) Differentially expressed genes involved in fat associated pathways.
